# Psychocentricity and participant profiles: implications for lexical processing among multilinguals

**DOI:** 10.3389/fpsyg.2014.00557

**Published:** 2014-06-30

**Authors:** Gary Libben, Kaitlin Curtiss, Silke Weber

**Affiliations:** ^1^Department of Applied Linguistics and Department of Psychology, Brock University, University of CalgarySt. Catharines, ON, Canada; ^2^Language Research Centre, University of CalgaryCalgary, AB, Canada; ^3^Department of Applied Linguistics, Brock UniversitySt. Catharines, ON, Canada; ^4^Department of Linguistics, University of CalgaryCalgary, AB, Canada

**Keywords:** psychocentricity, psycholinguistics, lexical processing, multilingualism, Chernoff faces, facial profiles, P3 experiments

## Abstract

Lexical processing among bilinguals is often affected by complex patterns of individual experience. In this paper we discuss the psychocentric perspective on language representation and processing, which highlights the centrality of individual experience in psycholinguistic experimentation. We discuss applications to the investigation of lexical processing among multilinguals and explore the advantages of using high-density experiments with multilinguals. High density experiments are designed to co-index measures of lexical perception and production, as well as participant profiles. We discuss the challenges associated with the characterization of participant profiles and present a new data visualization technique, that we term Facial Profiles. This technique is based on Chernoff faces developed over 40 years ago. The Facial Profile technique seeks to overcome some of the challenges associated with the use of Chernoff faces, while maintaining the core insight that recoding multivariate data as facial features can engage the human face recognition system and thus enhance our ability to detect and interpret patterns within multivariate datasets. We demonstrate that Facial Profiles can code participant characteristics in lexical processing studies by recoding variables such as reading ability, speaking ability, and listening ability into iconically-related relative sizes of eye, mouth, and ear, respectively. The balance of ability in bilinguals can be captured by creating composite facial profiles or Janus Facial Profiles. We demonstrate the use of Facial Profiles and Janus Facial Profiles in the characterization of participant effects in the study of lexical perception and production.

In this paper, we present a psychocentric view of language representation and processing, one that claims that, fundamentally, language representations have their reality in patterns of cognitive processing (Derwing, 1973). We claim that the psychocentric perspective is particularly relevant to the study of language processing in multilinguals in general and in modeling of the mental lexicon of multilinguals in particular. Tapping psychocentric effects requires the ability to triangulate among language perception ability, production ability, and individual participant properties. We have found that high density experimental paradigms such as those employed by Libben et al. (2012a,b) can capture these effects within an integrated experimental framework and that the evaluation of participant profile effects can be augmented through data visualization techniques such as the ones we present in this paper.

## The psychocentricity of language

Language ability contains an in-built paradox. On the one hand, it is something that is shared among members of a speech community. On the other hand, it is something that we possess as part of our individual cognitive states and capacities. The depth and complexity of this paradox becomes apparent when we consider the meanings of the apparently simple terms such as *share* and *individual*.

Members of a speech community *share* a language. The meaning of the word *share* in this context is of course different from its meaning in sentences such as “They share a chocolate bar” or “They share a taxi.” In both of these cases, there is a well-defined external entity (i.e., the chocolate bar or the taxi) that is referred to. A language is different. Except for its codifications in grammatical descriptions or dictionaries, a language is not a well-defined external entity, but rather a generalized construct that results from the abilities and behaviors of individual community members.

This brings us to the term *individual*. Language resides in the minds of individuals. However, we also know that the possible variation in individual characteristics of language representation and processing in the mind are constrained. Decades of research on language disturbance as a result of damage to the brain and fMRI studies with unimpaired populations have offered substantial support to the view that our language behavior is both linked to and constrained by common features of brain anatomy and physiology (see Pulvermüller, 2005).

These commonalities provide the context and constraints within which individual differences and the effects of individual language experience can play a role in shaping the synchronic character of an individual's language ability. However, both the constraints and the abilities reside in individuals and, thus, it is the individual that constitutes the fundamental object of psycholinguistic inquiry. This is essentially the psychocentric perspective (Libben, 2010; Libben and Weber, 2014).

The psychocentric perspective on language processing affects the ways in with we think about what it means for members of a speech community to *share* a language. Taking lexical knowledge as an example, the psychocentric perspective claims that it is not the case that *English*, as a language, has these or those words in its vocabulary. Rather, it is members of the community that have these or those words, individually, in their vocabularies. Because new words are acquired throughout the lifespan and because their specific characteristics, both structural and semantic, are influenced by patterns of individual experience, the psycholinguistic characteristics of words will differ from one person to the next.

## Psychocentricity and the shift toward great complexity in experimental design and analysis

The psychocentric perspective on language representation and processing, by definition, increases the complexity of the psycholinguistic enterprise by opening the doors to individualized notions of language unit and linguistic structure. However, this is very much in line with developments in the field as a whole. In a great many domains of psycholinguistic research we have seen a shift from small, highly controlled, factorial experiments to ones that embrace both participant and stimulus complexity (Libben et al., 2012a,b). A good deal of this shift is made possible by new statistical techniques such as mixed effects modeling (Baayen, 2008; Baayen et al., 2008), and by the much more widespread use of computationally implemented models to both advance claims about language representation and process and also to test the predictions that correspond to those claims. Because computationally implemented models receive their support or lack thereof as a result of their performance rather than through their representational transparency in traditional box-and-arrow flowchart models, they can much more easily incorporate complexity.

The developments outlined above make it possible to incorporate the complexity associated with a psychocentric perspective into the practice of psycholinguistic experimentation. The embracing of complexity marks a significant shift in the design of psycholinguistic studies. For example, in the domain of lexical processing, which is our focus in this paper, simplifying strategies have traditionally been a pervasive feature of experimental designs. Accordingly, the differences that might exist among experimental participants were seldom core features of experiment reports (Libben and Jarema, 2002).

## Psychocentricity and multilingualism

The majority of the world's population speaks more than one language (Grosjean, 2012). And, considerable evidence has shown that bilinguals show patterns of performance that differ from those of monolinguals in their respective languages (e.g., Gollan et al., 2005; Ivanova and Costa, 2008; Gollan and Goldrick, 2012). These findings underline the point brought forward by Grosjean (1989) that we cannot simply assume that a bilingual, even a balanced bilingual, has two sets of monolingual linguistic abilities in one brain. What follows from this is that, in the case of bilingualism, the state of the language system will be even more individualized because it must accommodate, within a single cognitive architecture, potentially disparate linguistic systems. Moreover, the balance of those systems will vary greatly depending on the particular experience of the multilingual. More often than not, a monolingual's ability in his or her languages will not be balanced. This underlines how, indeed, we cannot consider bilinguals to have two sets of language ability in one brain.

Another factor that is relevant to the characterization of the language ability of the multilingual is that, whereas monolinguals typically show comparable language production and comprehension abilities, this is not always the case for multilinguals. This fact has particular relevance to the value of high density psycholinguistic paradigms, such as the P3 paradigm that we discuss below, in psycholinguistic research with multilinguals. Finally, there is an additional reason why the language ability of multilinguals must be seen psychocentrically. That is that, except for cases in which the languages of a multilingual are acquired early in life, the language system of the multilingual will be in greater flux than that of a monolingual. This is perhaps most evident in the domain of lexical processing which we consider below.

## Implications for the mental lexicon and lexical processing

We see the mental lexicon as a theoretical construct that refers to the store of words in the mind, the organization, and the abilities and processes involved in employing words in language comprehension and production. Thus, if a multilingual is in possession of a single mental lexicon, whatever differences might exist between the two or more languages of a multilingual will need to be accommodated within a single cognitive system for lexical comprehension and production (Libben, 2000; Libben and Goral, in press). For the most part, these interlingual lexical differences will be most evident for multimorphemic words. And, these, rather than their simpler monomorphemic counterparts constitute the norm (Libben, 2007). Although we often consider words as atomic representations that are stored in memory and retrieved for the purposes of language comprehension and production, most words of English and other languages are not composed of a single unit of meaning, but rather contain two or more constituent morphemes.

The facts that most people are multilingual and most words are multimorphemic have important consequences for our understanding of the dynamic nature of the mental lexicon and lexical ability. Throughout our lives we learn new words. And, as a consequence of learning these new words, we develop new associations among words and, from those associations, complex networks of word families. An educated native speaker of English will, throughout adulthood, encounter many words that he or she has never seen or heard before. Most of these words will be multimorphemic and thus morphological knowledge can be used to guess at the meaning on the basis of analogy with existing morphological patterns. When new words are acquired, morphological patterns are expanded and in some cases new morphological families are created. Thus, it is most appropriate to see the mental lexicon not as static store of individual representations (an image that is perhaps inherited from the metaphor of a language dictionary in the mind) but rather as a dynamic system of knowledge and knowledge processing that can be influenced by experience both in quantitative and qualitative ways. Quantitative changes may involve the expansion of vocabulary in childhood and adulthood as well as a possible contraction of vocabulary size as a result of disuse and aging (Goral et al., 2007).

Typically, a very dramatic jump in the size of an individual's vocabulary will occur when a second language is acquired. Indeed, perhaps the most dramatic difference between the mental lexicon of a monolingual and the mental lexicon of a bilingual is that, in the latter case, the individual simply knows many more words. According to Aitchison (1987), a typical speaker of English will know about 75,000 words. There is no evidence that learning an additional language (outside of cases of language attrition) is accompanied by a diminution of that number. Therefore, one might expect that a high functioning balanced bilingual would, *ceteris paribus*, have a vocabulary size of considerably more than 75,000 words. A high-functioning polyglot may have many more.

The qualitative effects upon the mental lexicon of the acquisition of a new language are substantial. Firstly, if we consider translation-equivalent lexical items to be special cases of synonymy, a speaker of multiple languages will possess an enriched network of synonymy. And, there may be multiple structural consequences. One language may have grammatical gender, the other might not. One language may have interfixes, the other might not. The languages of the multilingual may differ in their morphological headedness. And, they may differ in their patterns of prefixation and suffixation.

If we assume that the potential interlingual differences described above must be accommodated within a single cognitive system, it follows that the functional organization of the mental lexicon of multilinguals will have substantially greater complexity of structure and function than that of a monolingual. And, it is this complexity that gives rise to the need to track and analyze individual effects in the psycholinguistic study of lexical processing in multilinguals.

By definition, the language experience of multilinguals will be more heterogeneous than that of monolinguals. Thus, they will differ from each other more. On the one hand, this creates challenges to generalizability of results to broader populations, as it can be claimed that a multilingual is, by nature, *sui generis.* On the other hand, if our goal is to understand the ways in which the state of the mental lexicon and lexical ability are driven by experience, it is exactly the heterogeneity of experience that we should be seeking out and seeking to analyze and understand. Programs of psycholinguistic experimentation that embrace the kinds of complexity that this involves will need to employ methodologies and analyses that are both robust in the face of participant heterogeneity and at that same time sensitive enough to make use of the subtleties that they reveal.

These issues have been associated with longstanding debates in psychology. The distinction between a focus on the individual (the idiographic approach) and a focus on the group (the nomothetic approach) has been at the core of debate in both the experimental and clinical psychological literature since the introduction of the dichotomy in the late nineteenth century (see Robinson, 2011 for a review). Although the idiographic-nomothetic distinction has often been framed as an issue of appropriate sample size, the psychocentric approach that we describe does not lead to a favoring of small samples. Rather, the psychocentric approach intersects the idiographic-nomothetic dichotomy with respect to the matter of the locus of language as a theoretical construct. The psychocentric perspective claims that it is not external stimuli that have linguistic properties *per se*. It claims that linguistic properties reside as part of complex, dynamic systems within an individual. Thus, a conceptualization of language stimuli as “out there in the world” may be an example of what Alfred North Whitehead first coined as “the fallacy of misplaced concreteness” (Flynn, 1997).

## The use of high density experiments to bring together participant, perception, and production data

In the discussion above, we framed the challenge of psycholinguistics in terms of the psychocentric perspective. Within this perspective, the goal of psycholinguistic experimentation is to tap into the many facets of the dynamic system of language ability. Libben et al. (2012a) outlined the functional architecture of an experimental paradigm designed to achieve this goal in a manner that brings together measures of both language perception and production. The term they use, *P3*, for this paradigm refers to the key components: *participant, perception, and production*. As such, it falls within the category of what we term high density experiments, those that yield a rich set of dependent variables.

In its most general format, the P3 task can be considered to be a type of dictation task. Dictation is a highly integrative activity that has deep roots in both the practice of second language teaching and second language learning. It has long been considered to be a reliable indicator of overall language ability because it brings together almost all elements of language cognition. The dictation task is sensitive to the manner in which both comprehension and production can be integrated. The reason for this is that if the dictation stimulus is not perceived easily, it will be difficult to write because the writer is unsure of the nature of the stimulus and also because unfinished and ongoing perceptual processes must be carried out simultaneously with production processes. And, the greater the extent to which the writer has automatized production processes, the faster they will be able to be carried out, creating a lower level of demand upon cognitive resources. The dictation task was at the center of discussion in the language testing literature of the 1960s and 1970s (e.g., Carroll, 1961; Oller, 1971) and has been used successfully in psycholinguistic experiments, particularly those which have focused on sub-elements of the writing process (e.g., Frisson and Sandra, 2002).

**Figure 1:**
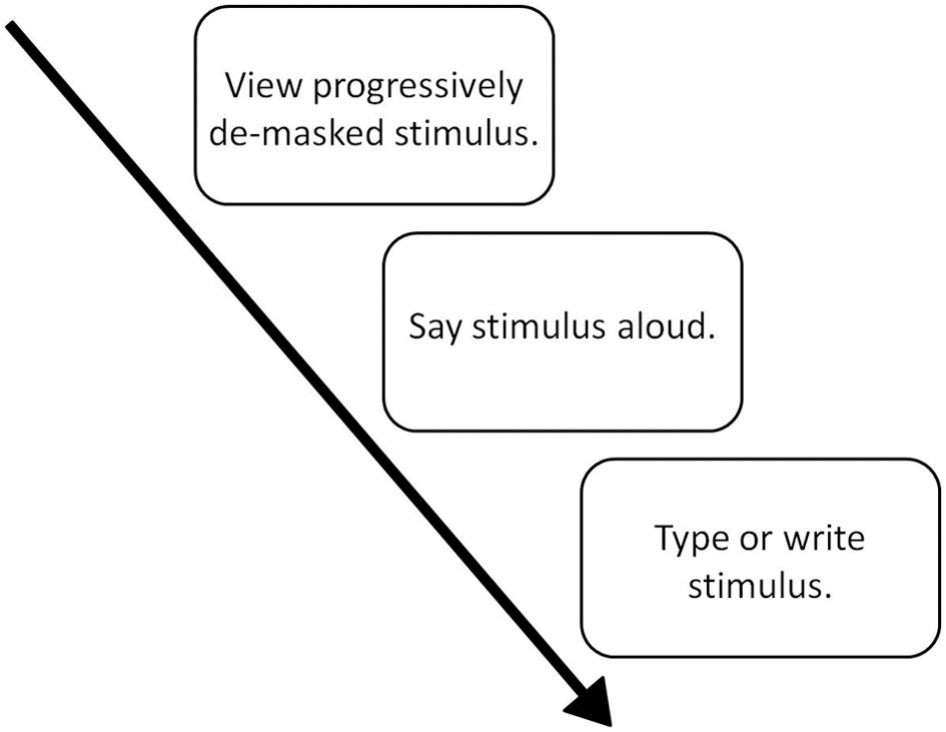
**The P3 paradigm**.

The basic structure of a P3 experiment, as shown in Figure 1, involves three core components (1) the viewing of the stimulus (2) the oral production of the stimulus, (3) the writing of the stimulus. We discuss each of these, in turn, below.

### Viewing the stimulus in a P3 experiment

The stimulus viewing component of the P3 paradigm was designed to probe lexical processing by building upon established techniques. We developed a variant of the progressive de-masking technique developed by Grainger and Segui (1990). This technique requires that participants recognize a linguistic stimulus as soon as possible. Stimulus presentation in the progressive de-masking technique differs from the more common format of word recognition paradigms in which a visual stimulus appears suddenly, and often for a very brief duration. In the progressive de-masking technique, on the other hand, a stimulus is presented over an extended period of time, emerging, as it were, from a fog. It is imperceptible at first, and then becomes slowly visible over a period of up to 3 s.

In the implementation that we have created for this task using PsyScope X for the Mac, the “out of the fog” effect is created by alternating stimulus presentation with a pattern mask of cross-hatches (#####) for 18 cycles of 300 ms (ms). In the first cycle, the stimulus is presented for only 16 ms and the pattern mask, immediately following is presented for 284 ms to create the total cycle duration of 300 ms. This proportion of stimulus duration to mask duration shifts by 16 ms in favor of stimulus duration in each successive cycle. From the perspective of the participant, cycles are continuous. So the participant simply perceives the stimulus becoming stronger and the pattern mask becoming weaker. This continues until the participant responds or until the 18 cycles have been completed. In the final cycle, it is the stimulus that is presented for 284 ms and the pattern mask that is presented for only 16 ms.

The entire presentation sequence takes almost 3 s. In practice, however, participants' response times to multimorphemic words are in the range of 1000–2500 ms. This fact is important because it demonstrates how the progressive de-masking technique is at once an online recognition task, but at the same time not one that requires extremely fast reaction-time-like responses. Thus, it has in-built applicability for use with second language learners as well as participants of different ages.

Libben et al. (2012a) also report the ways in which the progressive de-masking technique can incorporate masked priming as part of the stimulus presentation. The masked priming technique (Forster and Davis, 1984) has as its key motivation, the need in psycholinguistic experiments to block the participant's ability to make strategic guesses about the nature of a stimulus target on the basis of their conscious analysis of the prime. It is claimed that because masked priming durations are very brief (often less than 40 ms) there is insufficient time for such strategies to be developed. The progressive de-masking paradigm allows prime stimuli to be used in the early cycles of stimulus presentation. Our testing of the paradigm has revealed partial repetition prime differences when partial repetition primes have been incorporated into the initial two cycles (16 and 32 ms) of a progressively de-masked presentation.

The key strength of progressive demasking as a visual word recognition paradigm in comparison to lexical decision is that it does not require the presence of non-words in the experiment and does not require a metalinguistic judgment. Nevertheless, it is not without its drawbacks. Ferrand et al. (2011) compared the progressive demasking technique (using a button press response) to lexical decision and naming in a megastudy. The results of this research showed progressive demasking to be particularly sensitive to the visual characteristics of stimuli (e.g., length and the initial letter of the target string). In addition, however, the authors also reported that progressive demasking showed slightly greater sensitivity to semantic factors in comparison to lexical decision. It could indeed be the case that these observations are related to the effectiveness that Libben and Weber (2014) report for progressive demasking (across a number of versions) in the study of semantic transparency in English compounds. Here, sensitivity to semantic factors is exactly what is desired of the paradigm. Because compound words served as stimuli in this study, it may also be the case the sensitivity to length effects and initial segment effects were minimized. Compounds vary percentage-wise in length much less than monomorphemic words do, and, as Ferrand et al. note, initial segment effects are greatest for short words (Balota et al., 2004). Compound words are, by virtue of their structure, typically among the longer words of a language.

In our view, the progressive demasking task, despite the drawbacks noted by Ferrand et al. (2011) has particular applicability in a bilingual setting (see Lemhöfer et al., 2008) because it is not prone to influence by the composition of the non-words set of stimuli required in a lexical decision task. Moreover, as we have noted above, the distinction between words and non-words among bilinguals working in their non-native language, and in particular, second language learners, cannot be assumed to be identical to the word-nonword distinction for native speakers of a language.

### Oral stimulus production in a P3 experiment

In the original progressive de-masking technique presented by Grainger and Segui (1990), participants indicated their recognition of a progressively de-masked word by pressing the response time key. Libben et al. (2012a) modified the technique so that participants indicate their recognition by saying the word aloud as quickly as possible. This provides the opportunity to assess response time through a voice key and also enables the recording and analysis of phonetic properties of the response. This modification of the classical progressive de-masking technique is necessary to enable the entire P3 paradigm to function as a type of dictation task. As we discuss below, the P3 paradigm has two fundamental variants. The first is a one-participant variant in which the same person sees the stimulus, says it, and then writes it. The second is a two-participant variant in which the first participant sees the word and says it aloud and the second participant writes it. By modifying the response type to an oral response, therefore, we create a situation in which the one-participant variant and the two-participant versions of the paradigm are exactly comparable terms of event structure. In addition, we gain the opportunity for speech analysis within the paradigm.

### Writing of the stimulus in a P3 experiment

The third component of the P3 paradigm focuses on the written production component of the overall dictation task. Here too Libben et al. (2012a) built upon existing techniques. A number of studies within the last decade have demonstrated the manner in which the analysis of writing and typing can be used to address key questions in psycholinguistics in general and in the study of lexical processing in particular. Kandel et al. (2006) and Alvarez et al. (2009) have shown that by analyzing handwritten responses it is possible to gain insight into the effects of syllable structure and syllable boundaries (see also Kandel et al., 2011). Kandel et al. (2008) also demonstrated handwriting effects in the morphological domain by contrasting truly suffixed and pseudo-suffix words of French. This research, together with the typed response research by Will et al. (2006) as well as by Sahel et al. (2008) demonstrates the manner in which online written production is influenced by the morphological structure of words as well as other variables relevant to the organization within the mental lexicon such as constituent and word frequency.

The fact that such factors emerge in the analysis of writing serves to remind us that features of words such as morphological structure have their fundamental reality in the minds of language users and are revealed through their activity. In a writing task, participants are not surprised by stimuli. Rather, they are revealing, through their writing, the nature of their internal representations. If a participant pauses at syllable and morpheme boundaries as has been found in the above studies, this demonstrates that such structures serve to organize the chunking of their motor activity in production.

Our implementation of the writing component within the P3 paradigm has both handwritten and typewritten variants. The typewritten version produces data that are more easily analyzed. However, those data are less rich than those available to the net through the analysis of handwriting, in which we currently have the ability to measure both within-letter and between-letter durations, as well as (depending on the hardware employed) measures such as pen jitter and pen pressure.

### Capturing participant profiles through online questionnaires and stimulus evaluation

The P3 technique, particularly in the single-participant version, produces a participant profile by enabling the analysis of word recognition latencies, oral production characteristics, and written production characteristics. In our implementation we have augmented these sources of evidence with two additional components.

The first is the use of a participant questionnaire that documents the participant's background on a number of variables as well as his or her experience with other languages. Because the P3 paradigm incorporates, by design, a writing component, it was natural to employ this technology in the acquisition of questionnaire data. This yields not only data regarding the actual answers to questionnaire items but also writing duration data for the questionnaire as a whole.

The P3 design also incorporates a post online experiment component in which participants are asked to rate the stimuli that they have seen along a number of dimensions. For multimorphemic words, these include overall word frequency, perceived age of acquisition, and semantic transparency ratings for morphological constituents. Together these constitute individualized measures for stimulus predictor variables that are typically used in the analysis of the effects of stimulus characteristics upon language performance.

### Single participant and dual participant variants of the P3 technique

In our view, the P3 paradigm opens up an opportunity to compare language processing in traditional psycholinguistic laboratory settings to a somewhat more ecologically valid context in which individuals are interacting. To be sure, a two-person dictation task is nothing like a conversation. However, we suggest that the ability to contrast one-participant and two-participant versions opens up a number of opportunities for the study of second language and multilingual processing. The paradigm makes it possible to break down the components of the overall experiment so that the possible effects of individual variants can be examined. It is perhaps appropriate to consider the single participant version to be the base version. This is the one in which a single participant sees a progressively de-masked stimulus, says it aloud, and then writes it. The effect of having seen the stimulus oneself and of having said the stimulus aloud oneself can be isolated by substituting, for those components, a version in which the oral stimulus is presented by a computerized text-to-speech program. In this case, participants are engaged in a single task: writing to computerized dictation.

By substituting computerized dictation for human dictation in the two-participant version, and by varying the first language background of the first speaker, it is possible to employ the P3 paradigm to measure second-language speech comprehensibility, as operationalized by written production accuracy, written production latency, and written production duration.

### Illustrative example: the use of the P3 technique in the study of the semantic transparency of english compound nouns

Libben and Weber (2014) employed this adaptation of the P3 technique in a study of sematic transparency in English compounds. They employed a core stimulus set of 40 noun-noun English compounds that differed in terms of the semantic transparency of their compound constituents. Thus, a compound such as *sailboat* was classified as transparent–transparent (TT) because the meanings of both the compound constituents *sail* and *boat* are preserved in the meaning of the whole word. At the other extreme, a compound such as *humbug* was classified as opaque-opaque because neither the meanings of *hum* or *bug* are preserved in the meaning of *humbug*. Between these two extremes were opaque–transparent compounds such as *nickname* and transparent–opaque compounds (TO), such as *jailbird* (TO). These compounds had been studied in a lexical decision task by Libben et al. (2003) and they thus offered the opportunity to compare the P3 technique for the same set of stimuli. The core stimulus set is presented in Table 1.

**Table 1:** **The core compound stimulus set employed by Libben and Weber (2014)**.

**TT**	**OT**	**TO**	**OO**
Bedroom	Chopstick	Cardshark	Deadline
Coalmine	Crowbar	Doughnut	Dingbat
Daylight	Dashboard	Heatwave	Fleabag
Doorbell	Godchild	Jailbird	Hallmark
Farmyard	Jackknife	Oddball	Hogwash
Fencepost	Nickname	Shoehorn	Humbug
Paintbrush	Pothole	Slowpoke	Ragtime
Rosebud	Shortcake	Sourpuss	Rugrat
Sailboat	Strawberry	Spoilsport	Stalemate
Schoolboy	Sunfish	Staircase	Windfall

*Compounds are classified as TT, transparent–transparent; OT, opaque–transparent; TO, transparent–opaque; and OO, opaque–opaque*.

Ninety-three native speakers of English participated in four versions of a P3 experiment. The versions contrasted individual vs. dyadic formats and whether or not a naming response or a button press response was made.

Before the main experiment, participants filled out a questionnaire on their language background and some demographic information. We have used the data derived from this questionnaire as input to the creation of the visual participant profiles that we present in section The Use of Facial Profiles in Monolingual and Multilingual Processing of the present report. Such questionnaire data are of course particularly valuable in cases in which the P3 experiment is conducted in participants' non-native language. Indeed, one of the advantages of the technique for the study of multilingual performance is that it has its roots in the second language testing.

**Figure 2:**
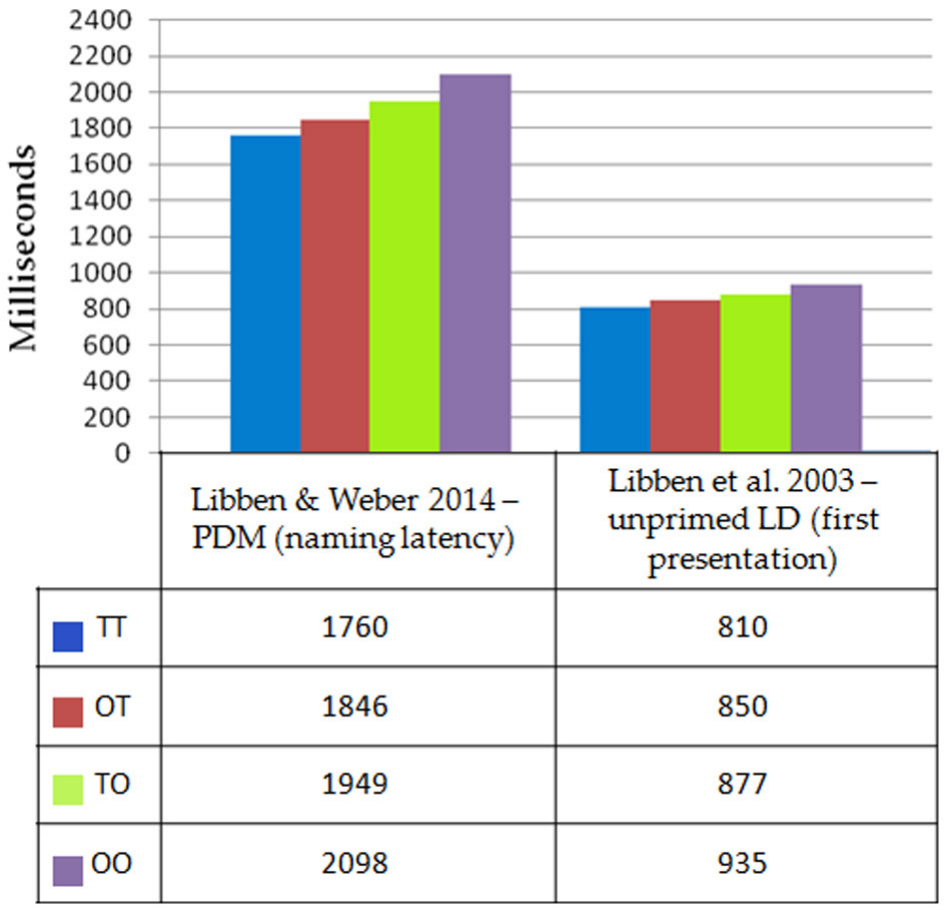
**Comparing the progressive demasking results of Libben and Weber (2014) and the lexical decision results of Libben et al. (2003) for four types of compounds: transparent–transparent, TT; opaque–transparent, OT; transparent–opaque, TO; and opaque–opaque, OO**.

All versions of the P3 paradigm showed the same data pattern for the four types of compounds. Thus, the results from the individual version, the interactive version, and the visual version for the analysis of progressive demasking latencies were merged. The resulting latency patterns are shown in the leftmost portion of Figure 2. As can be seen in Figure 2, the progressive demasking latencies were greatest for opaque-opaque compounds and least for TT compounds. This pattern accords with the lexical decision latency patterns obtained by Libben et al. (2003) and which are shown for comparative purposes in the right section of Figure 2. The difference in the scales of the response times for the two paradigms results from the nature of the progressive demasking paradigm in which, as described in section Viewing the Stimulus in a P3 Experiment above, stimuli are presented in incremental durations over a three second period.

In the second part of the P3 procedure, following the progressive demasking response, participants in the Libben and Weber (2014) study were asked to type the stimulus word. The results of the calculation of typing latencies yielded a pattern of results that supported the conclusion that typing latencies are affected by morpheme boundaries and that semantic transparency affects those latencies. These results accord with those of Sahel et al. (2008) who reported both these effects.

**Figure 3:**
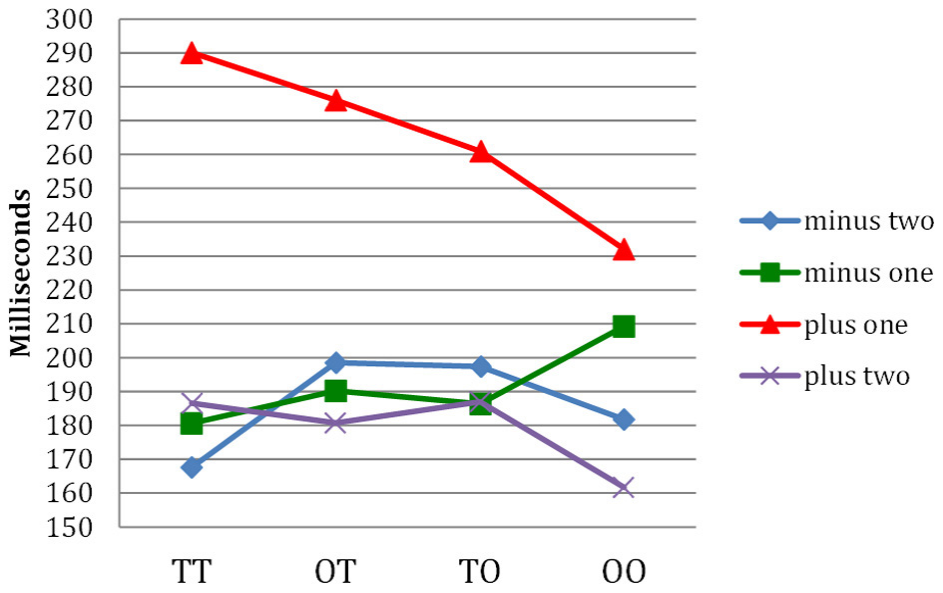
**Letter typing times in milliseconds for four types of compounds investigated by Libben and Weber (2014).** The morpheme boundary is considered to be *position zero*. Position *minus two* is thus two letters before the boundary, position *minus one* is the letter before, and position *plus one* is the letter immediately following the morphemic boundary. Thus, for the compound sailboat, positions minus two to plus two would comprise the letters “i,” “l,” “b,” and “o.” The time taken to type the letter at position *plus one* (e.g., the “b” in *sailboat*) is interpreted as the time taken to pause at the morpheme boundary.

Figure 3 shows the pattern of typing latencies found by Libben and Weber (2014). For all compound types, the letter immediately following the morpheme boundary (the “plus one” condition) shows the greatest typing times. Those times were greatest for the TT compounds and least for the opaque-opaque compounds. Thus, the typing component of the paradigm supports the view that, in TT compounds, the full compound is “chunked” in terms of its constituent morphemes. This seems to be much less the case for the opaque-opaque compounds. It is worthy of note that the progressive demasking component and the typing component of the P3 technique target two distinct facets of compound transparency phenomena. The progressive demasking component targeted overall ease of processing. The typing component targeted the manner in which the stimuli differ in terms of the extent to which they can be characterized as being internally structured.

### Summary: P3 experiments provide a means by which participant profiles can be created

The P3 paradigm that we have presented above is essentially a recombination of existing psycholinguistic methodologies. The advantage of this type of recombination for the study of second language processing and multilingual processing is its high density as a technique. By high density, we mean the ability to generate a very rich set of data for each individual. As we discussed at the outset of this paper, experimentation with second-language users and multilinguals requires that researchers capture the heterogeneity that typically exists within the linguistic ability and linguistic performance of this group. The P3 technique enables the creation of participant profiles by enabling the triangulation of perception, oral production, and written production. This is particularly important for second-language users and multilinguals because there are often imbalances across those domains that are much larger than those that would be expected for native speakers and monolinguals.

The creation of a participant profile is also supported by the two adjunct procedures that we discussed above, namely the questionnaire and the off-line stimulus evaluation. These are of course not unique to the P3 paradigm. In any experiment with second-language users or multilinguals, an extensive language use and language background questionnaire will be of considerable value. Typically, however, employing the characteristics of participant profiles in the analysis of online data is less easy. One reason for this is that there is typically more questionnaire data collected that can be used in the analysis of online performance. Another reason is that when we do use such data as predictor variables in experiments, they are often used not as full profiles but rather as individual predictors. To a large extent, this problem can be overcome by bringing a multitude of variables together through a principal components analysis and then using the values of those principal components as predictor variables. In the section below, we present a supplement to such statistical techniques that involves a simple visual recoding of participant variables to create profiles.

## The use of facial profiles in monolingual and multilingual processing

Throughout this paper, we have foregrounded the role played by the individual as the fundamental unit of psycholinguistic investigation. A perspective such as this creates somewhat of a paradox when we try to incorporate participant characteristics as predictor variables in psycholinguistic experiments. The reason for this is typically that we do this one variable at a time. Yet, we know that these variables must be considered as integrated components of an individual. If we take the term *individual* relatively literally, as that which cannot be divided, it seems reasonable to seek a means by which we can be aided in understanding the manner in which participant characteristics are indeed within a participant. In this section, we present a data visualization technique that we consider to be supportive of the psychocentric perspective on language processing and which can be valuable as a data analysis heuristic.

Our technique is based on the computer-generated faces developed by Chernoff (1973). In this approach, Chernoff reasoned that because humans, as a species, have a special aptitude for recognizing and analyzing small differences in facial structure and expression, it might be possible to use faces to code the values of many more variables than could normally be represented in a graph and have the values and relations among these variable more easily perceived by the researcher or the reader of data reports.

In our view, it is noteworthy that this technique, developed over 40 years ago, has been perhaps more talked about than actually used, despite the creativity of the approach and its potential application in a variety of domains (see De Los Reyes et al., 2013). It thus seemed to us that the basic insight that Chernoff brought to the domain of data visualization is worthy of both further consideration and further development. And, it may well be possible that a variation on the original Chernoff (1973) contribution could serve as an extremely valuable adjunct to high density paradigms such as the P3 paradigm discussed above.

In their original form, Chernoff faces used a multitude of facial characteristics to recode variables. These features included the following: size of face, length of nose, vertical position of mouth, curvature of mouth, width of mouth, separation of eyes, slant of eyes, eccentricity of eyes, size of eyes, position of pupils, vertical position of eyebrows, slant of eyebrows, and size of eyebrows.

**Figure 4:**
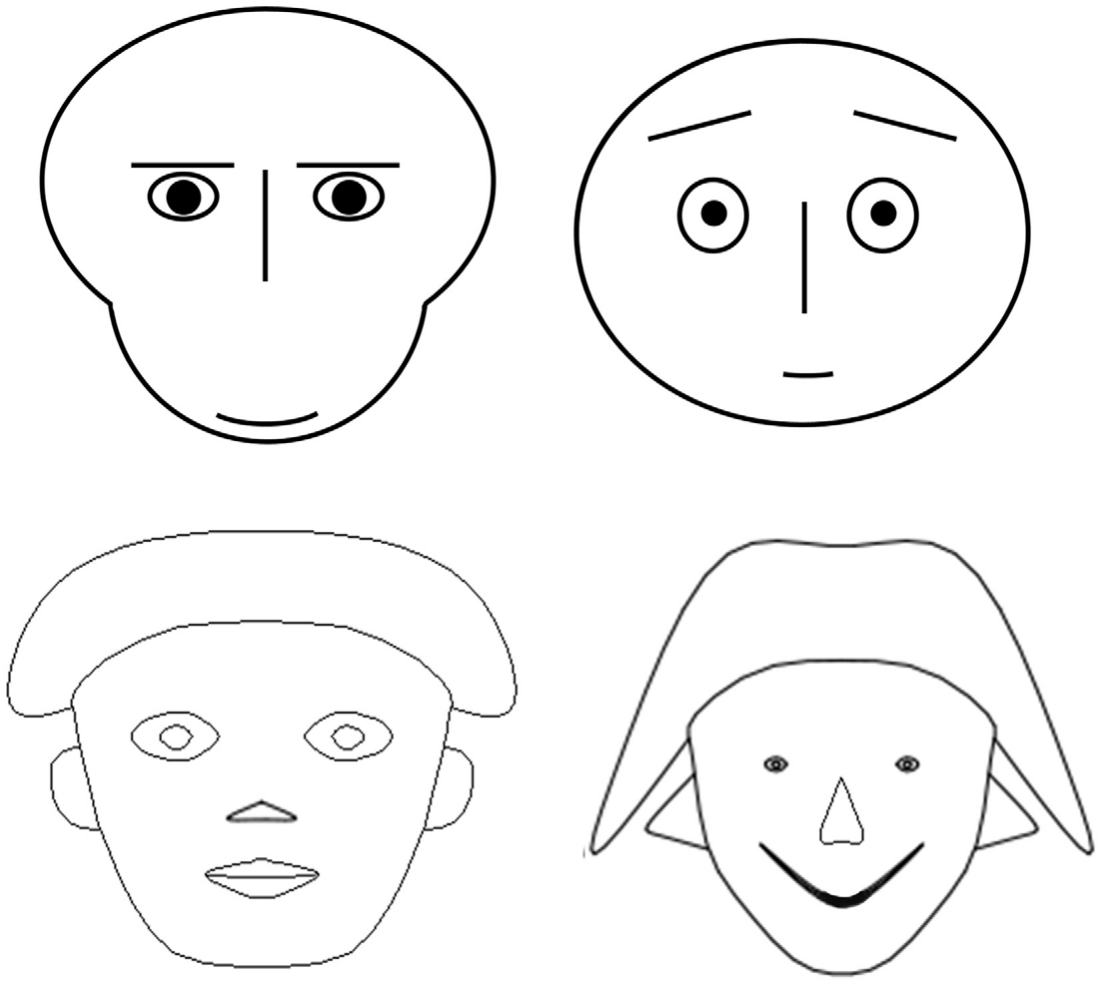
**Examples of Chernoff style faces**.

Samples of faces in the Chernoff style are shown in Figure 4. At first blush, they seem to provide the perfect opportunity to instantiate a psychocentric approach to the analysis of psycholinguistic data. By embedding participant characteristics such as those obtained in the P3 questionnaire described above, it should be possible to graphically demonstrate the manner in which elements of a participant profile are indeed components of an integrated cognitive system.

There are however, achieve challenges to the use of classical Chernoff faces. The first is that their complexity often does not always allow them to the discrimination for which they were designed (Morris et al., 2000).

The second challenge is relatively straightforward: the manner in which Chernoff faces were originally created makes it difficult to transparently recode values into faces so that the researcher has the feeling that this is simply a reversible data transformation.

The third challenge is considerably more substantial. As is likely evident from a quick perusal of the original Chernoff facial features listed above, and as is shown also by Morris et al. (2000), facial features differ substantially in their salience. The human face recognition system prioritizes certain features over others, giving them a greater weight. This is a specific instance of the larger issue in the utilization of Chernoff's original insight. That insight was essentially that by mobilizing the human face recognition system, a very powerful, biologically driven, system would be able to detect small quantitative differences and interpret them as qualitative differences. This is at once the chief strength of the technique and its chief weakness. The biological face recognition system can be simply too powerful. Mobilizing it is, in many ways, like inviting a gorilla into your living room.

Our goal was to address all of these challenges while maintaining the advantages of using faces to code participant profiles. We reasoned that the first step was to ensure that there was a relatively transparent data recoding mechanism and that facial features would be relatively balanced for salience. To achieve this goal, we turned the faces sideways to produce two-dimensional profiles. Doing this solved one problem immediately. When seen in profile, it is no longer the case that a face has two eyes and two ears but only one nose and one mouth. This, at least to some extent, addressed the problem of facial feature imbalance. It also had the effect of “toning down” the powerfulness of facial features by reducing their affective impact on the viewer (in a manner that is comparable to the way in which profile faces in Ancient Egyptian hieroglyphic inscriptions have diminished affective impact).

**Figure 5:**
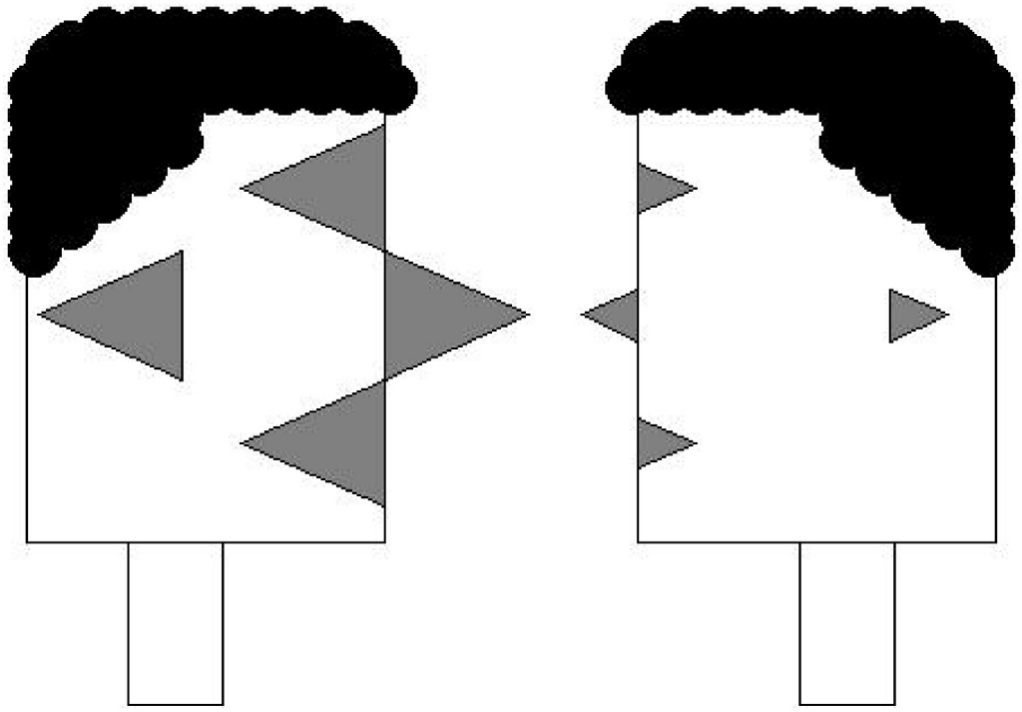
**Examples of Facial profiles showing maximum variable size and minimum variable size**.

The second innovation that we employed was to implement a transparent data recoding algorithm by simply recoding data values as the size of equilateral triangles and then by using those equilateral triangles as features of a facial profile. The use of triangles has two advantages: First, it is not uncommon to see eyes, nose, and ears stylized as triangular representations. Second, the use of triangles opened up the option of coding other variables as variations of those triangles. The first option employs degrees of shading. The second option is to change the shape of a triangle, while keeping the area constant. Thus, equilateral triangles can be changed to isosceles triangles with the same area. In Figure 5, examples of two Facial Profiles is provided. These examples show the maximum feature size as well as the minimum feature size in our current implementation.

Below, we demonstrate how Facial Profiles can be derived from simple datasets. We begin with an example taken from the performance of five sample bilinguals participants drawn from the P3 experiment reported in Libben and Weber (2014). In Table 2, their performance is shown with respect to four characteristics, their self-assessed reading, writing, speaking, and listening abilities.

**Table 2:** **Self-assessed reading, writing, speaking and listening abilities for five participants on a 7-point scale**.

**Participant**	**Reading**	**Writing**	**Speaking**	**Listening**
1	4	5	6	6
2	6	7	7	7
3	7	7	7	7
4	7	5	6	6
5	6	6	7	7

**Figure 6:**
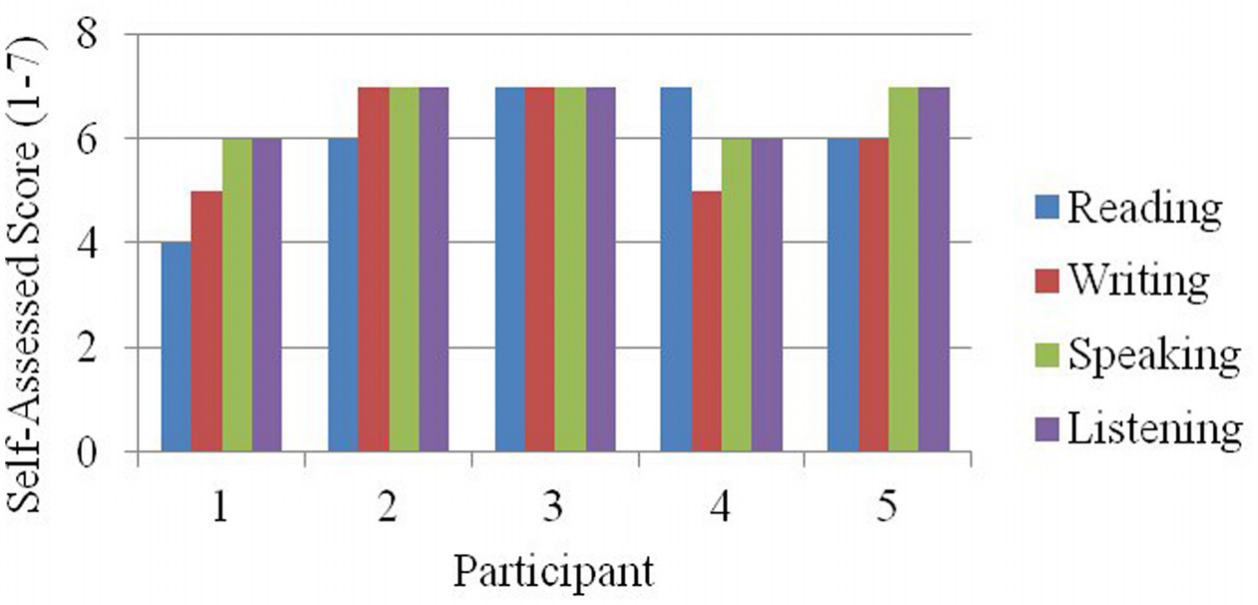
**Self-assessed reading, writing, listening, and speaking ability for the five participants described in Table 1.** This bar graph recoding of the data from Table 1 is the first step in the development of visual participant profiles.

To demonstrate the evolution of participant profiles from the data in Table 2. We begin with a bar graph (Figure 6) that is simply a graphic rendering of the data in Table 2.

**Figure 7:**
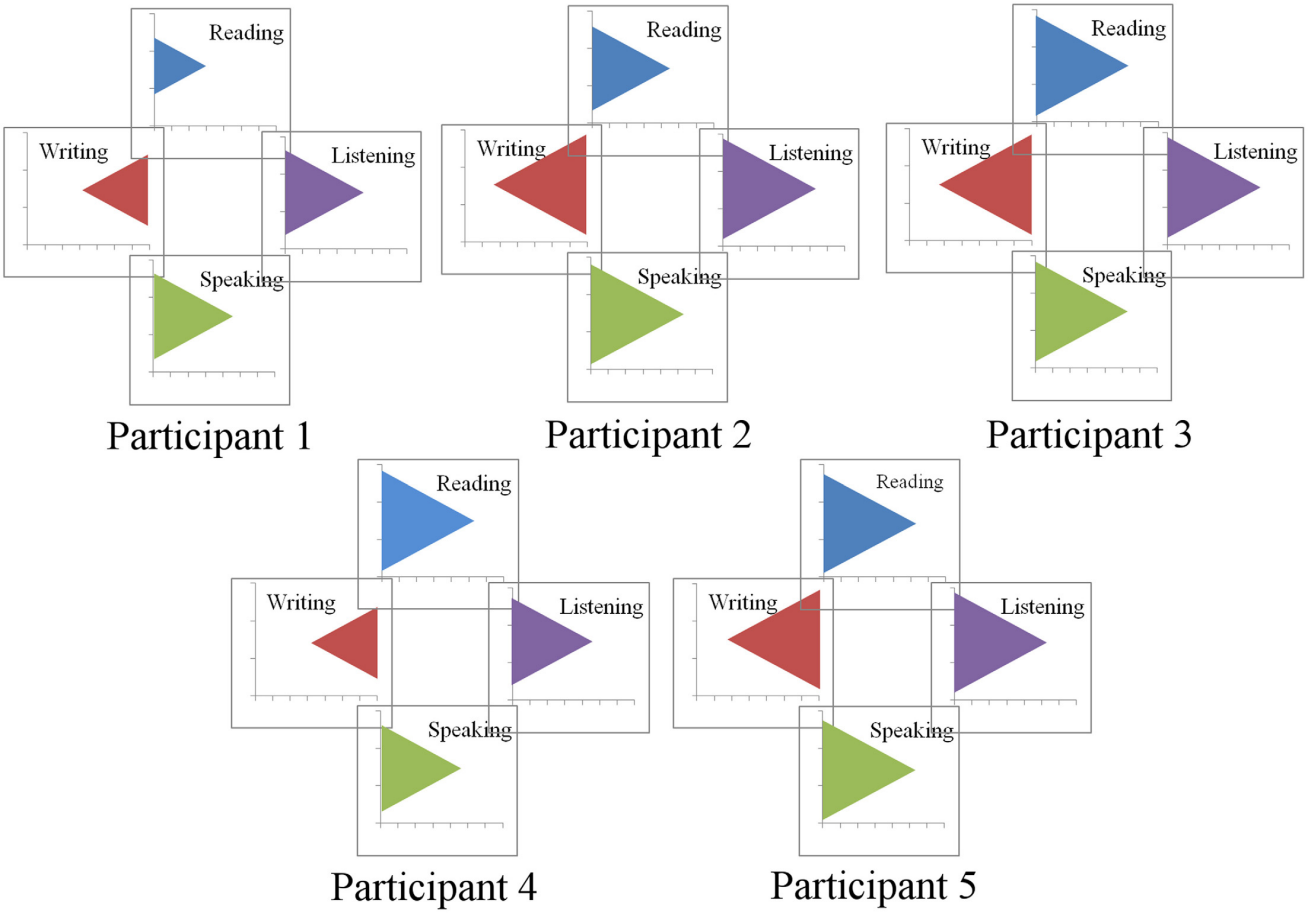
**Recoding of bar graph into relative triangle sizes.** This is the second step in the development of visual participant profiles.

**Figure 8:**
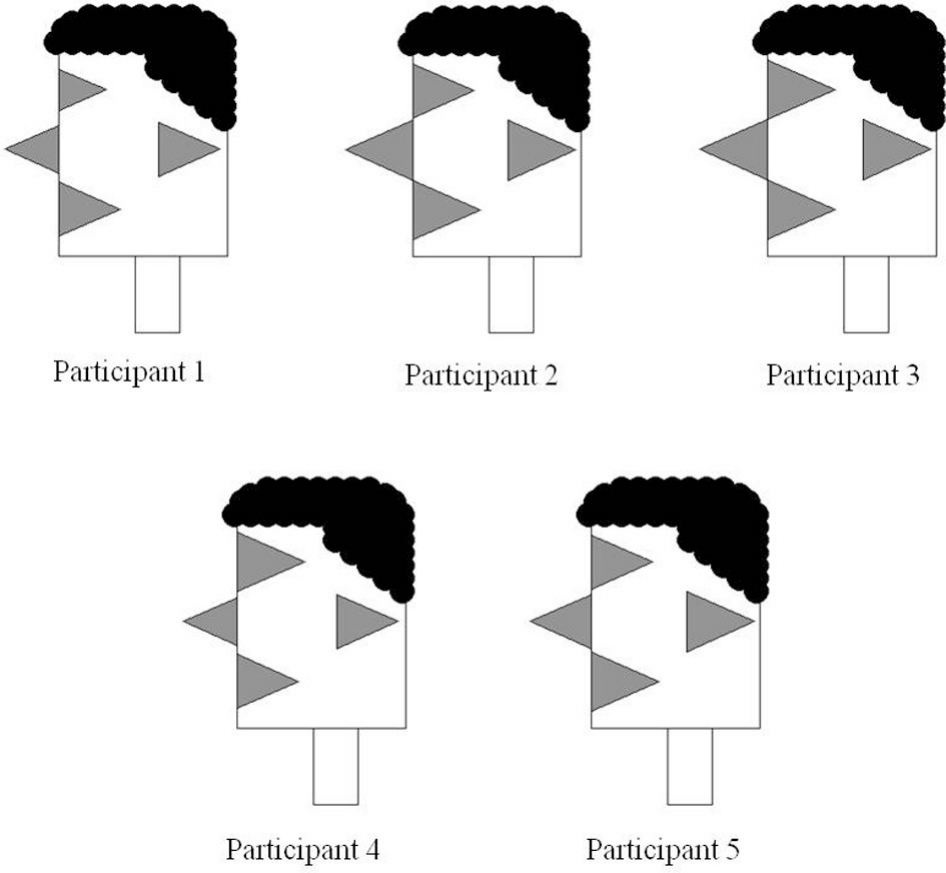
**Encasing the triangles from Figure 5 into facial profiles enables their perception as facial features.** Recoding of bar graph into relative triangle sizes. This is the third and final step in the development of visual participant profiles.

In Figures 7, 8, the power of participant profiles to bind and unite individual feature values is demonstrated. Figure 7 was created by recoding the bar graph in Figure 6 so that the heights of the bars are represented as areas of equilateral triangles. Those triangles are arranged into the configurations in which they will appear as the participant profiles.

**Figure 9:**
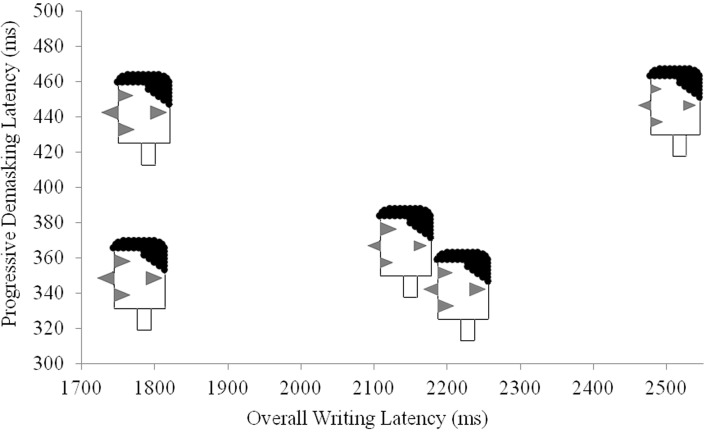
**Participant profiles used to replace participant data points on a scatterplot**.

The representations in Figure 8 are identical to those in Figure 7, except that the triangles are monochromatic and encased in participant facial profiles. As such, the representations in Figure 8 can be analyzed in terms of their individual feature characteristics. In addition, they can also be used as complex units that may interact with other variable configurations within an experiment. In Figure 9, we demonstrate how data from these five participants can be used in conjunction with performance data in a P3 experiment that, for example, plots progressive de-masking latencies against overall writing latencies. The data shown in Figure 9 are exactly the data obtained for these five participants in the experiment reported by Libben and Weber (2014) and which is described in section Illustrative Example: The Use of the P3 Technique in the Study of the Semantic Transparency of English Compound Nouns above. As can be seen in Figure 9, the faces essentially replace what would be individual data points in a scatter plot, while at the same time demonstrating component characteristics of those individual data points.

**Figure 10:**
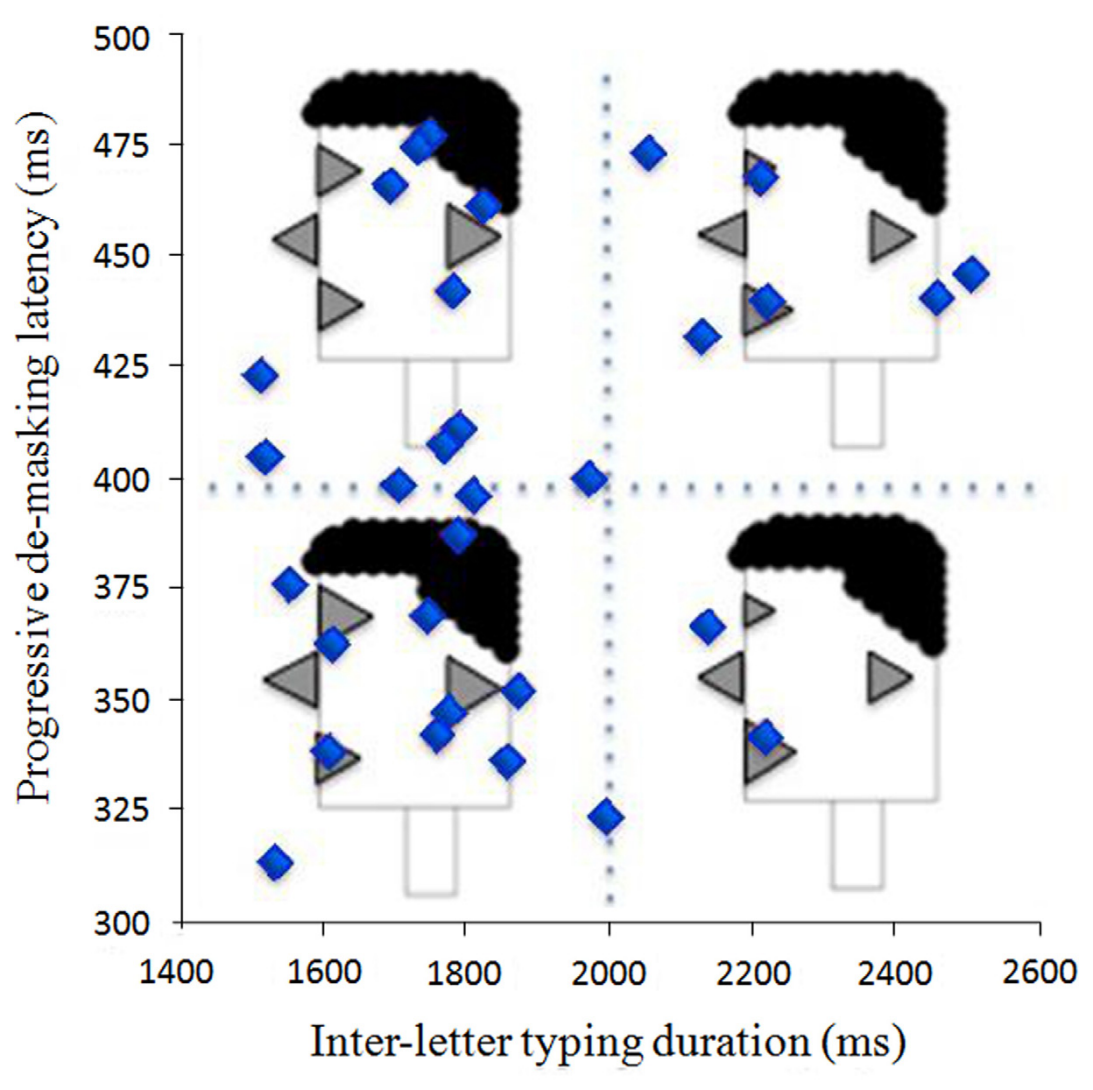
**Using facial profiles to code average values within a quadrant of graph space**.

The approach shown in Figure 9 is effective when a small number of data points are involved. However, its usefulness decreases as the number of points to be plotted increases. The main reason for this is simply that an increase in the number of data points to be plotted will require that each facial profile be decreased in size so that they can all fit within the plot. As a result, the features, and hence their relative sizes, will be more difficult to see. A solution to this challenge is presented in Figure 10. Here, an entirely different approach is taken. Instead of using facial profiles to characterize individual participants, the plot has reverted to a standard scattergram, with each point representing an individual participant. Data for Figure 10 are drawn from the progressive demasking latencies and typing latencies for 32 participants from the Libben and Weber (2014) who had performed both tasks and who had filled out the participant profile questionnaire at the outset of the experiment. The plot space has been divided into quadrants. For each quadrant, a single facial profile is constructed. These facial profiles are set as essentially watermarks upon which the plot is displayed. They key feature of such quadrant facial profiles is that they do not represent individuals. Rather, they represent the average features for each profile variable of all the participants (i.e., dots) in that quadrant. We see this approach as offering benefit in aiding in the understanding of how participant characteristics (both literally and metaphorically) map onto performance variables in a P3 experiment.

### Janus facial profiles and interactive facial profiles

The final two types of facial profiles that we discuss bring us back to issues of multilingualism and the interactive nature of the P3 paradigm respectively.

As we have claimed throughout this paper, multilinguals have particularly complex participant profiles, which must be taken into consideration in the understanding of performance data. So far, we have used facial profiles to code participant characteristics in a single language. It is, of course, possible to use the profiles to code participant characteristics in more than one language. In order to make more variables available, and in order to iconically represent bilingual characteristics, we have created what we term *Janus facial profiles*, named for the Roman god Janus. Janus is characterized as the god of beginnings and transitions and is traditionally depicted as having two faces, one looking to the past and the other to the future.

The Janus facial profile offers a convenient means of depicting bilingual features while maintaining the notion of individual integrity. Janus profiles can be used in any of the configurations described above, i.e., as representations of individuals and as representations of group characteristics.

**Figure 11:**
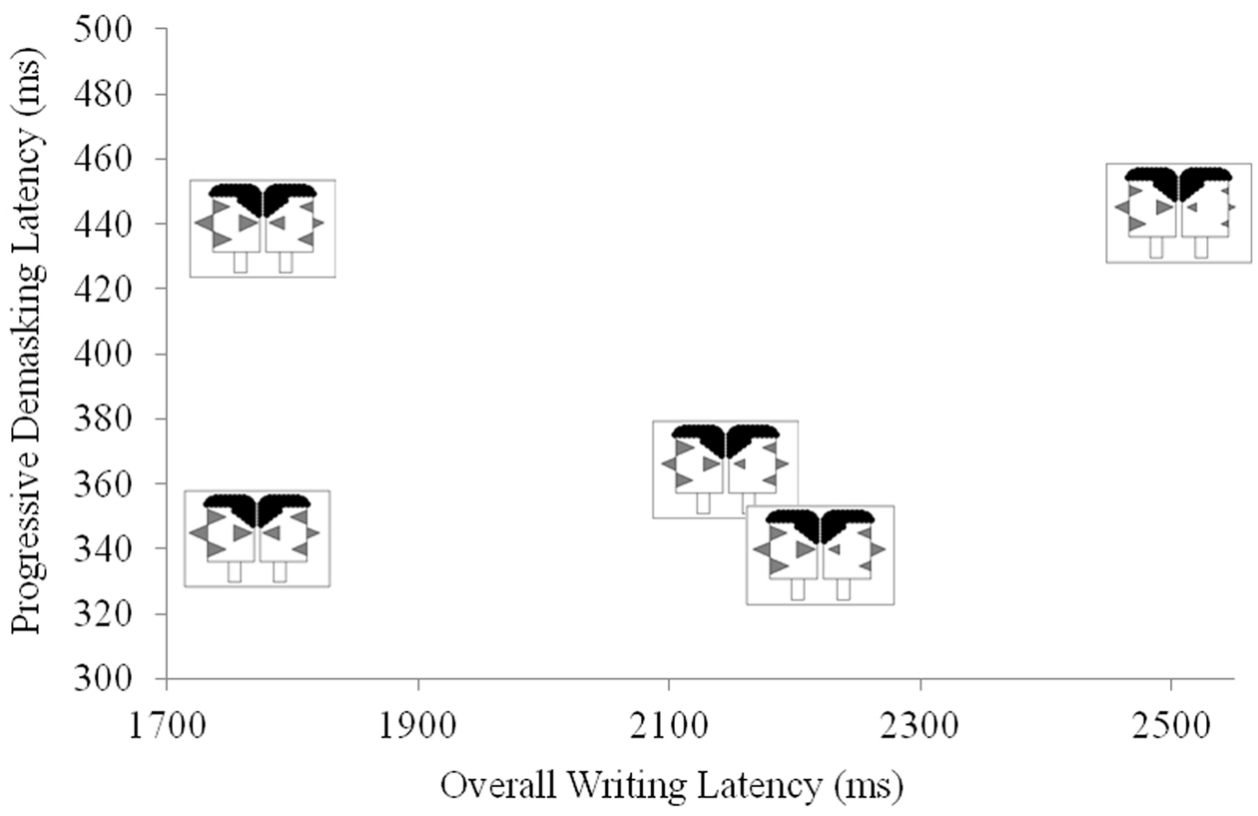
**Using Janus facial profiles to code individuals' first and second language abilities and their relations to on-line performance in a P3 experiment**.

The Janus faces also enable the final variant of facial profiles that we discuss. By using two faces in juxtaposition, it is possible to use facial profiles to represent participant dyads, such as those that comprise the pairs in the two-participant versions of the P3 experiments we describe above. In this way, participant facial profiles can be used as a convenient means of inspecting the extent to which participant pairs in interactive P3 experiments share selected characteristics. In Figure 11, we demonstrate the use of Janus Faces by adding bilingual information into the data presented in Figure 9 above. The Janus faces in Figure 10 replicate the left-facing profiles in Figure 9 and add reading, writing, listening and speaking ability in the right-facing profiles.

### Inter-participant variability and intra-participant variability

**Figure 12:**
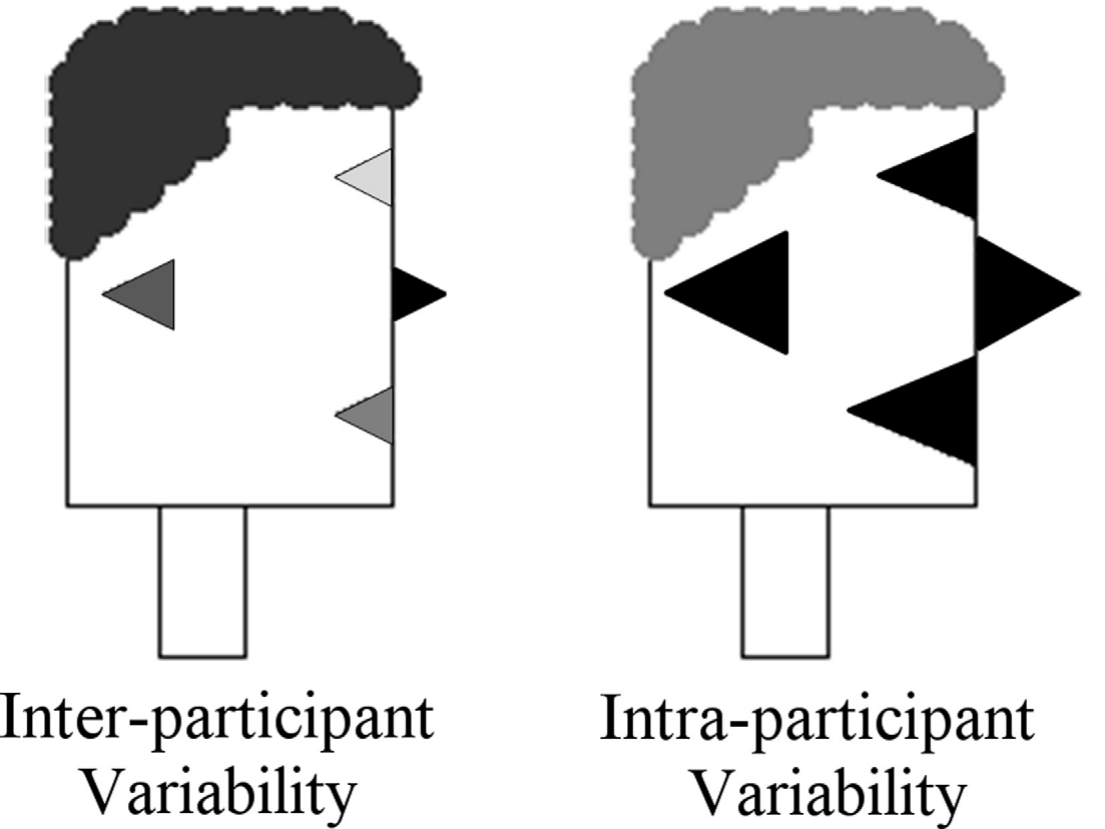
**Inter-participant variability (Left) coded by feature shading and intra-participant variability (Right) coded by hair shading**.

In the sections above we have provided examples of how Participant Profiles can be used to encode scores or values associated with individuals and which are coded as the size of individual facial features. In Figure 10, we have also shown how group averages for specific variables can be encoded as facial features. In our design of participant profiles and their instantiation in R, we have also developed the means by which both inter-participant variability and intra-participant variability can be represented. We have used feature color/grayscale shading to core variation, so that fully color saturated features represent a standard deviation of zero. Shading becomes lighter as the standard deviation increases. Examples of this are shown in the left panel of Figure 12. The Participant Profile in this panel is identical to the type of group profile shown in Figure 10 in that the size of the facial features represent group means for the variables. In this case, however, standard deviations are included so that we are able to also view the relative variability for each feature across the individuals in the group.

For Participant Profiles that encode individuals on a graph (e.g., Figures 9, 11), it can also be valuable to be able to encode not only the sizes of individual features, but also the relative variability among features. We have implemented this type of intra-participant variation in the shading of hair color. Here too, smaller variability is associated darker shading and higher variability (i.e., lesser density) is associated with lighter shading. An example of this is shown in the right panel of Figure 12.

### Summary: facial profiles enable participant characteristics to the integrated into the presentation and interpretation of P3 results

The examples of Participant Profiles that we have presented above demonstrate a new means by which participant characteristics can be linked to patterns of experimental performance in paradigms such as that P3 paradigm described above. As we have discussed above, our elaboration of the approach pioneered by Chernoff (1973) is designed to build upon human face recognition ability while constraining the manner in which it can affect the interpretation of multivariate data. By using faces in profile, we addressed some of the challenges of feature salience. The use of triangles was designed to homogenize the shape of each feature and to ensure full translatability of the variable values into triangle size. The approach also enabled the translation of data density (leptokurtic vs. platykurtic distributions) into grayscale or color saturation. The ability to both encode multivariate means and standard deviations, the ability to link variables thematically to facial features, and the ability to mobilize the interpretive power of the human face recognition system distinguishes Facial Profiles from other multivariate data visualization techniques such as radar graphs. Finally, in the development of Participant Profiles, we have retained the ability to turn equilateral triangles into isosceles triangles with the same area, but a different shape. This creates the ability to code for more values for each face, resulting in a potential total of 26 values that can be coded within each Janus Profile.

## Conclusion

We have presented a psychocentric perspective on language processing that embeds the linguistic nature of language structures within a psychological matrix. We have focused on the mental lexicon and lexical processing in multilinguals and have claimed that the nature of lexical representation and processing among multilinguals will be greatly shaped by experience across the lifespan.

We have claimed that high density experiments such as the P3 paradigm that we discuss are particularly advantageous in the study of lexical processing among bilinguals because they offer a rich set of dependent variables and have the capacity to simultaneously capture features of both language perception and production as well as participant profiles. To aid in the understanding of how those participant profiles interact with other aspects of experiment performance, we have proposed a data visualization technique that is based on the faces developed by Chernoff (1973).

The methodological and data visualization techniques that we propose can have application in many domains of language performance. In particular, though, we see them as having value in the study of lexical processing. Decades of psycholinguistic research have focused on the manner in which elements of the mental lexicon are connected. Indeed, the entire program of masked priming research beginning with Forster and Davis (1984) has served to show the dimensions along which words in the mental lexicon are linked to one another. We have seen that they are linked along all dimensions-semantic, morphological, and phonological. And, research on bilingual lexical processing has shown that the bilingual (and by extension multilingual) lexicon is also richly networked. If we combine this observation with the fact, discussed above, that the lexicon is in a dynamic state throughout the lifespan, then new representations, i.e., newly acquired words, are not simply added to a list. Rather they are integrated into a network. As a result of that integration, the network changes character with each newly acquired component. Understanding this integration is the next key challenge for psycholinguistic research in lexical processing. We suggest that the techniques we have presented have potential to aid in addressing that challenge.

### Conflict of interest statement

The Review Editor Dr. Mary Grantham O'Brien declares that, despite being affiliated to the same institution as author Silke Weber, the review process was handled objectively and no conflict of interest exists. The authors declare that the research was conducted in the absence of any commercial or financial relationships that could be construed as a potential conflict of interest.
